# The Effects of Alcohol Consumption on Recovery Following Resistance Exercise: A Systematic Review

**DOI:** 10.3390/jfmk4030041

**Published:** 2019-06-26

**Authors:** Nemanja Lakićević

**Affiliations:** Sport and Exercise Research Unit, Department of Psychological, Pedagogical and Educational Sciences, University of Palermo, 90144 Palermo, Italy; nemanja.lakicevic@unipa.it; Tel.: +39-3515881179

**Keywords:** strength, training, muscle mass, muscle function, performance

## Abstract

Background: The aim of this manuscript was to describe the effects of alcohol ingestion on recovery following resistance exercise. Methods: A literature search was performed using the following database: Web of Science, NLM Pubmed, and Scopus. Studies regarding alcohol consumption after resistance exercise evaluating recovery were considered for investigation. The main outcomes took into account biological, physical and cognitive measures. Multiple trained researchers independently screened eligible studies according to the eligibility criteria, extracted data and assessed risk of bias. Results: A total of 12 studies were considered eligible and included in the quantitative synthesis: 10 included at least one measure of biological function, 10 included at least one measure of physical function and one included measures of cognitive function. Conclusions: Alcohol consumption following resistance exercise doesn’t seem to be a modulating factor for creatine kinase, heart rate, lactate, blood glucose, estradiol, sexual hormone binding globulin, leukocytes and cytokines, C-reactive protein and calcium. Force, power, muscular endurance, soreness and rate of perceived exertion are also unmodified following alcohol consumption during recovery. Cortisol levels seemed to be increased while testosterone, plasma amino acids, and rates of muscle protein synthesis decreased.

## 1. Introduction

Resistance exercise (RE) is a commonly practiced modality of physical exercise used by both amateurs and elite athletes [1]. RE is a type of exercise that has gained a lot of interest over the past two decades, specifically for its role in improving athletic performance by developing muscular strength, power and speed, hypertrophy, local muscular endurance, motor performance, balance, and coordination [2]. While non-athletes use it to simply develop muscular physique, professional athletes engage in RE to enhance their athletic capabilities in various sports [3]. Variables such as exercise intensity, exercise frequency, load, number of sets and repetitions, rest periods and training volume can be manipulated in order to maximize RE induced effects in terms of muscle hypertrophy and strength [4]. Physiological and psychological constraints leading to a reduction in physical or mental performance can be classified as fatigue which is a phenomenon that has protective role in human physiology [5]. Exercise is a potent stimulus with respect to altering homeostatic variables which triggers adaptive reactions that counter the metabolic changes and repair the structural damage caused by the previous training session [5]. The stressful effects of RE can temporarily impair athlete’s performance [6]. Therefore, the speed and quality of recovery are absolutely essential for the high performance athlete and, if done correctly, optimal recovery can lead to numerous benefits training and upcoming competition [7]. The main purpose of recovery is to restore physiological and psychological processes, so that the person engaging in vigorous exercise can repeat training sessions at an appropriate level [7]. It is also typically dependent on the nature of the exercise performed and any other outside stressors that the athlete may be exposed to [7].

Certainly, one of the unnecessary stressors during recovery phase is alcohol (ALC) consumption [8,9]. Worldwide, alcohol is the most commonly used psychoactive drug; it is estimated that each adult person consumes, on average, about 4.3 L of pure alcohol per year [10]. In the current era, consumption of alcohol is increasing exponentially in Western society [11,12,13] and it is common knowledge that alcohol can permeate virtually every organ and tissue in the body, resulting in tissue injury and organ dysfunction [14]. Alcohol consumption results in hormonal disturbances that can disrupt the physiological ability to maintain homeostasis and eventually can lead to various disorders, such as cardiovascular diseases, reproductive deficits, immune dysfunction, certain cancers, bone disease, and psychological and behavioral disorders [14]. In terms of post exercise recovery, acute alcohol ingestion reduces muscle protein synthesis in a dose-and time-dependent manner, after the cessation of exercise stimulus [8]. Alcohol does this mainly by suppressing the phosphorylation and activation of the mTOR pathways, the crucial kinase cascade regulating translation initiation [8,15]. Concomitantly, alcohol increases the expression of muscle specific enzymes that are up regulated by conditions that promote skeletal muscle atrophy [8,16].

Emerging research provides new insights into the effect of alcohol consumption on post-exercise muscle recovery but more research is needed to determine how this relationship exists and establish the physiological mechanisms governing this response. Therefore, the aim of this review is to understand the effects of alcohol consumption during recovery, on muscle function, following RE.

## 2. Materials and Methods

Preferred Reporting Items for Systematic Reviews and Meta-Analyses (PRISMA) statement has been used to structure this manuscript.

### 2.1. Inclusion and Exclusion Criteria

Studies that meet the following criteria will be included or excluded in this systematic review.

### 2.2. Eligibility Criteria

When it comes to eligibility criteria, only articles written in English language and published in peer-reviewed journals have been considered during the search. There was no limit on publication date when it comes to article eligibility. Different formats of publications such as reviews, meta-analysis, abstracts, citations, scientific conference abstracts, opinion pieces, books, book reviews, statements, letters, editorials, non-peer reviewed journal articles and commentaries have been excluded. With respect to intervention, publications were included only if they used a specific measure of performance or biomarker, that considered recovery following RE and alcohol intoxication. Articles exploring recovery after endurance type of training have been eliminated.

### 2.3. Participants

All the analyzed participants were adults to whom an ALC intervention was administered following a bout of RE. Children were not considered for analysis. There was no limitation when it comes to age, gender, number of participants, and duration of intervention or follow up period. Furthermore, there was no limitation when it comes to training status.

### 2.4. Interventions

The interventions described in the eligibility criteria will be included in this review. The interventions aimed to understand the effects of ALC on biological, physical and cognitive measures following RE. According to the nature of the review different methodological approaches which evaluate similar outcomes will be considered.

### 2.5. Comparators

Comparators will be control groups (people not consuming ALC [NO-ALC]) if present or if cross-over designs will be adopted the intervention groups will act as controls after each wash-out period.

### 2.6. Outcomes

The primary outcome will be to understand the effects of ALC compared to the NO-ALC intervention. Such findings will be applied to all the biological, physical and cognitive measures retrieved.

### 2.7. Search Strategy

We used EndNote v. 8.1 software (Clarivate Analytics, Jersey, UK) for the article search. The papers have been collected through PubMed (NLM), Web of Science (TS), and Scopus using the string: ((“alcohol” AND “exercise*” and “recovery*”; “ethanol” AND “exercise” AND “recovery”; “alcohol*” AND “resistance training” AND “recovery”; “ethanol” AND “resistance training*” AND “recovery”; “alcohol*” AND “strength*” AND “recovery”; “ethanol” AND “strength” AND “recovery”; “alcohol*” AND “training” AND “recovery”; “ethanol” and “training” and “recovery”)).

### 2.8. Selection of Study Objects

**Figure 1:**
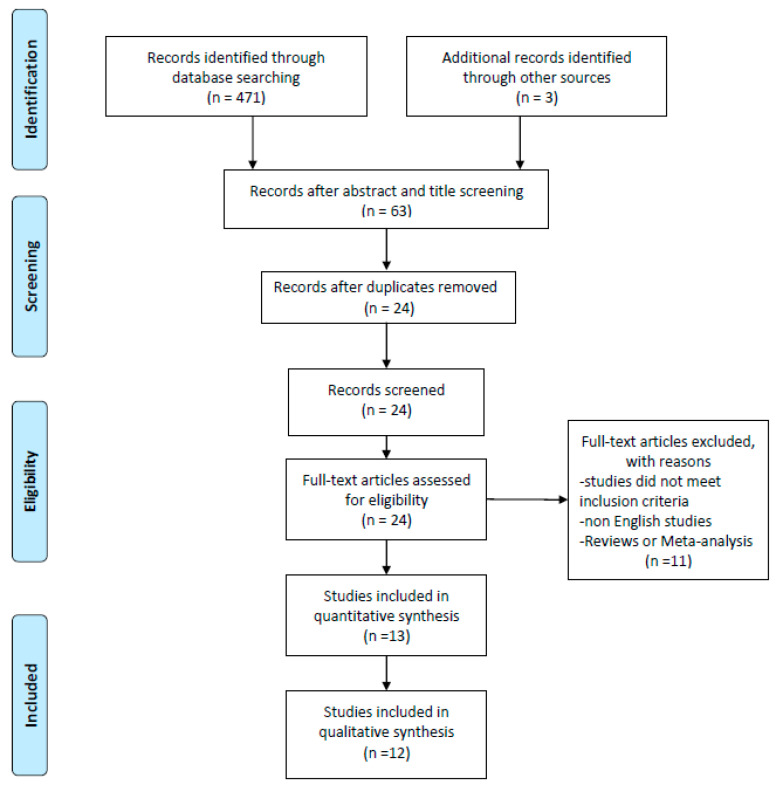
Prisma Flow Diagram

The article screening was carried out in a three-step process: title reading, abstract reading and full text reading, respectively. If any disagreements were noticed between the two investigators, a third one considered the current process independently and discussed the decision with the other investigators. Furthermore, investigators were not blinded to the manuscripts, study title, authors, or associated institutions during the selection process. Both qualitative and quantitative articles were included in the review. The screening processes have been summarized via PRISMA flow diagram (Figure 1).

### 2.9. Risk of Bias

Risk of bias for the included studies was assessed through Downs and Black checklist [17]. This tool is useful when evaluating the quality of original research articles in order to synthesize evidence for public health purposes. This checklist contains 27 ‘yes’-or-’no’ questions over five different domains. It offers both an overall score for study quality and a numeric score out of a possible 32 points. The five domains contain questions about study quality, external validity, study bias, confounding and selection bias, and power of the study.

Two independent researchers have completed the Downs and Black checklist of all included articles to determine the quality of each study. The maximum score a study can receive is 32, with higher scores indicating better quality. The studies were then divided into groups and marked as ‘high quality’ (score 23–32), ‘moderate quality’ (score 19–22), ‘lower quality’ (score 16–18) or ‘poor quality’ (<14) (Supplementary File S1 and S2). Kendall Tau correlation coefficient statistical method was used to determine inter-rater reliability. We used R statistical software (Bell Laboratories, Murray Hill, NJ, USA) version 3.6 to perform this analysis. Quality of evidence was determined by the study design and by Downs and Black score.

### 2.10. Data Synthesis

The critical information acquired from the included articles was extracted into Microsoft Excel for Macintosh, version 14.0 (Microsoft Corp, Redmond, WA, USA) spreadsheet. The most important characteristics of the studies, as author name and publication year, sample size, aim, alcohol dose, how this was mixed and administered, the study measures, the RE protocol adopted, the study design and the outcomes have been delineated in the tables while certain specifics about the particular study were described in a narrative manner.

## 3. Results

From a preliminary title and abstract search, a total number of 471 studies have been identified in the three screened databases. After the application of inclusion criteria on each article’s title and abstract, 63 records were considered eligible. Duplicates were removed leaving 24 studies for full text screening. After full text screening, two additional studies from the relevant bibliography have been added. Of the 24 studies analyzed, 10 were included for the final synthesis. Therefore a total number of 12 studies were included in the qualitative synthesis of this review article. The process of article inclusion has been synthesized in Figure 1. Risk of bias assessment was finalized through Downs and Black checklist for all included studies (Supplementary File S1 and S2). The mean score was 19 (range = 12–28). After performing the inter-rater reliability test via Kendall Tau analysis we detected the score of 0.46 which can be classified as moderate but significant (*p*-value 0.026). As previously stated, after comprehensive screening, files were split into different quality categories in accordance to predetermined quality criteria (Supplementary File S1 and S2).

In order to evaluate the effects of alcohol consumption on recovery following RE, the results have been summarized into three categories: (1) biological and (2) physical measures and (3) cognitive function.

Of the retrieved studies, 10 took into account at least one biological measure [9,18,19,20,21,22,23,24,25,26], 11 took into account at least one physical measure [9,18,19,20,21,22,23,24,25,27,28] and cognitive function only one [24].

The retrieved biological measures include creatine kinase (CK) [18,19,20,21,22,23,24,25], heart rate [19,26], lactate [19,26], blood glucose [9], urine measures [24], cortisol [19,21,24,26], testosterone [19,21,24,26], estradiol [26], sexual hormone binding globulin (SHBG) [21,26], leukocytes and cytokines [19,21,22], C-reactive protein (CRP) [24], plasma amino acids [9], intracellular signaling proteins and rates of muscle protein synthesis (MPS) [9] and calcium (Ca^2+^) [25]. The physical measures include force [18,20,21,22,23,24,25,27,28], power [19,21,24,28], muscular endurance [25], soreness [18,20,22,23,28] and rate of perceived exertion (RPE) [19,24,26]. The cognitive measures included a modified version of the STROOP test which evaluated time and accuracy of each response for congruent and incongruent stimuli. The alcohol dose provided to the participants in the included studies ranged between 0.6g/kg to 1.5g/kg. As defined by Kalinowski and Humphreys [29] a standard drink equals to 10 g of alcohol. Therefore, the alcohol dose provided to the participants, if we consider a man of 70kg, equals to 42 to 105 g of alcohol (4.2 to 10.5 standard drinks), which corresponds to 3 bottles of beer of 330 mL at 5% alcohol or a 370mL bottle of spirit at 37.5% alcohol, respectively. All the included studies adopted a cross-over research design. A summary of the retrieved studies is shown in Table 1.

### 3.1. Biological Measures

#### 3.1.1. Creatine Kinase

Eight studies included CK measurement [18,19,20,21,22,23,24,25]. All the retrieved studies showed that CK increases following each RE protocol in both ALC and NO-ALC conditions showing a time interaction with RE. However, when analysing interaction with the different conditions, no differences were shown between the ALC and NO-ALC condition for none of the retrieved studies. Clarkson et al. [20], have also correlated peak CK activity from the ALC and the NO-ALC condition and found a high correlation coefficient (*r*=0.95), whereas Paulsen et al. [25] have also stratified the findings for man and women finding again no differences between the two groups neither for time or treatment assessment. Such results, as also highlighted by each author of the included studies, demonstrate that ALC cannot be considered as a modulating factor for CK following RE and that the increases of CK are the result of muscle damage following the exercise bouts.

#### 3.1.2. Heart Rate

Two studies included measures of heart rate [19,26]. In both studies heart rate increased following the exercise intervention, however no difference was shown between the ALC and the NO-ALC condition.

#### 3.1.3. Lactate

Two studies included measures of lactate [19,26]. In both studies lactate increased following the exercise intervention, however no difference was shown between the ALC and the NO-ALC condition.

#### 3.1.4. Blood glucose

Only Parr et al. [9] evaluated the concentration of blood glucose. It has to be noted that Parr administered a concentration of alcohol in conjunction with either CHO or PRO. The results highlight a significant time and treatment interaction. Blood glucose concentration increased 0.5 and 4.5 h post intervention in the ALC-CHO group but not in the ALC-PRO or NO-ALC groups. Such findings demonstrate that blood glucose is affected by CHO but not ALC during recovery after RE.

#### 3.1.5. Urine Measures

Only Murphy et al. [24] included urine measures. Post-intervention urine output, nude mass and urine-specific gravity were measured. No differences were found for nude mass and urine-specific gravity between conditions. The ALC group had an increased total volume output overnight when compared to the NO-ALC group.

#### 3.1.6. Cortisol

Four studies included measures of cortisol [19,21,24,26]. In the study of Barnes et al. [19] the cortisol levels increased after 12h after treatment in both conditions, after which at 24h returned to baseline levels. A second rise in cortisol was seen at 36h under the ALC condition but not in the NO-ALC condition. Haugvard et al. [21] showed that no differences were shown between the two conditions at any specific time point after the intervention (12 and 24 h post-treatment). However, if the 12 and 24 h cortisol values were combined and averaged, these resulted to be significantly elevated only in the ALC condition at 24 h after the intervention. Murphy et al. [24] showed that a significant decrease post-match was followed by a significant increase at 16h post intervention. No difference was found in the levels of cortisol between the two interventions. However, a large effect size was found between the %change from 2 to 16 h post-match for the increase in cortisol response after ALC consumption. Vingren et al. [26] found that cortisol levels were not affected by ALC post intervention. Cortisol was elevated immediately after, after 20, 40, 60, 120, 140 and 300 min post-intervention in both ALC and NO-ALC conditions.

#### 3.1.7. Testosterone

Four studies included measures of cortisol [19,21,24,26]. In the study of Barnes et al. [19] no difference in the testosterone levels compared to baseline ware seen at any time point after the intervention in either two conditions (12-24-36 and 48 h after the intervention). In the study of Haugvard et al. [21] the levels of testosterone were not altered across trials neither for the ALC and the NO-ALC condition. Calculated free testosterone (testosterone/SHBG multiplied by a factor of 10) was not different between trials. However, if the measures at 12 and 24 h after the intervention were combined and averaged the levels of testosterone resulted to be lower only in the ALC condition after 24 h. Murphy et al. [24] showed that a reduction in testosterone was present after 2 h post-match followed by a significant increase 16h post-match. However, no differences were shown between the testosterone levels for the two conditions neither between %changes from 2 to 16 h post-match. Vingren et al. [26] found a significant effect for treatment for testosterone in which the levels were increased immediately and 140 and 300 min after the intervention for the ALC group, whereas it appeared to be decreased in the NO-ALC group between 60 and 300 min post-intervention. Free testosterone also seemed to be increased between 60 and 300 min post-intervention for both conditions.

#### 3.1.8. Estradiol

Only one study included measures of estradiol after ALC consumption [26]. The study indicates that the levels of estradiol were elevated immediately after and between 20 and 40 min after the intervention, when compared to baseline measures, in both groups, with no significant differences between the ALC and the NO-ALC group. The results underlie that ALC has no effect on estradiol during recovery from RT.

#### 3.1.9. Sexual Hormone Binding Globulin

Two studies included measures of SHBG [21,26]. In none of the included records the levels of SHBG seemed to be altered by ALC intake, neither by the acute bouts of exercise proposed by the two authors. ALC does not seem to be a modulating factor for SHBG following RE.

#### 3.1.10. Leukocytes and Cytokines

Three of the included studies included measures of leukocytes and cytokines [19,21,22]. Barnes performed analysis of total and differential leukocytes and found that total, neutrophil and monocyte concentration increased after the intervention but decreased to baseline values after 12h. However, no difference was present between the two conditions. Haugvard et al. [21] found no difference between conditions regarding the white blood cell, neutrophils or monocytes count. Following RE both conditions showed a sub-clinical leucocytosis 1h post-exercise. Levitt et al. [22] analysed inflammatory markers in women post-exercise, in particular TNF-α, IL-1β, IL-6, IL-8 and IL-10 before, at 5, 24 and 48h post-intervention.IL-10, IL-8 and TNF-α increased after the intervention in both groups. IL-6 and IL-1β remained unchanged over time for both conditions. No differences for cytokine was present between the ALC and the NO-ALC condition. ALC doesn’t seem to affect neither leukocytes nor cytokines after RE during recovery.

#### 3.1.11. C-reactive Protein

C-reactive protein was evaluated only in the study of Murphy et al. [24], in which however no significant difference was highlighted neither regarding time, when data was compared to baseline, neither regarding condition, when ALC and NO-ALC where compared. The findings indicate that post-match alcohol consumption did not unduly affect CRP markers of damage.

#### 3.1.12. Plasma Amino Acids

Plasma amino acids (AA) have been included only in the study of Parr et al. [9], who evaluated EEA, BCAA and leucine at 0, 1, 2, 4, 6 and 8 h after alcohol consumption following RE. It has to be noted that Parr administered a concentration of alcohol in conjunction with either CHO or PRO. The results were then compared to a control group who did not ingest ALC but consumed a single dose of 25 g of whey protein.

A significant effect for time and treatment were found. At all-time points the NO-ALC group had significantly higher levels of essential AA (EEA), branched chain AA (BCAA) and leucine compared to the ALC-PRO group. Both groups (the NO-ALC and the ALC-PRO) had at all-time points significantly higher levels of AA compared to the ALC-CHO group. No difference in the levels of AA compared to baseline was shown, at any time point, in the ALC-CHO group. Leucine, EEA and BCAA were elevated compared to baseline at 1 and 6 h post-ALC ingestion for the NO-ALC and ALC-PRO group. The data from the study of Parr et al. [9] indicates that ALC alone does not influence the levels of plasma AA, however can be a factor that limits the rise of blood concentration of AA following protein consumption.

#### 3.1.13. Intracellular Signaling Proteins and Rates of Muscle Protein Synthesis

mTOR, p70S6K, eEF2, 4E-BP1, AMPK, MuRF-1 mRNA and fractional synthetic rate of myofibrillar protein synthesis were analysed in the study of Parr et al. [9]. ALC and NO-ALC consumption modalities have been described in the previous subsection. mTOR^Ser2448^ phosphorylation was higher in all groups at 2h post treatment. However, mTOR phosphorylation in the NO-ALC group was higher than the ALC-CHO (76%) and ALC-PRO (54%) group at 2 and 8 h post-exercise. p70S6K phosphorylation was greater after 2h post-exercise compared to baseline only in the NO-ALC and the ALC-PRO groups. No differences were shown for the ALC-CHO group. eEF2 phosphorylation decreased below rest values at 2 and 8 h in the ALC-CHO and ALC-PRO groups. No differences were shown for the NO-ALC group at any time point.

No differences for time and condition were shown for 4E-BP1^Thr37/46^ or AMPK^Thr172^ phosphorylation. There were increases above rest in MuRF-1 mRNA at 2 h post- intervention with no differences between treatments. All values returned to baseline at 8h post- intervention.

Fractional synthetic rate of myofibrillar protein synthesis were increased above baseline for all groups from 2 to 8 h post-intervention. However, a hierarchical reduction was shown when data was compared to the NO-ALC group in the ALC-PRO (-24% compared to NO-ALC) and ALC-CHO (−38% compared NO-ALC and −18% compared to ALC-PRO) groups. Data suggests that ALC consumption impairs the response of muscle protein synthesis during recovery despite optimal nutrient provision.

#### 3.1.14. Calcium

Only one study has evaluated the effects of ALC on Ca^2+^ via blood sampling [25]. The authors report that the Ca^2+^ levels were similar before exercise for both conditions. A decrease of approximately 2% was observed following the exercise bouts. A further decrease was observed in the ALC condition, and the difference with the NO-ALC condition was significant only after the strength evaluation. Hypocalcaemia was not induced by ALC and no differences were shown for resting free Ca^2+^ levels indicating that free Ca^2+^ concentrations were not affected by alcohol per se.

### 3.2. Physical Measures

#### 3.2.1. Force

Nine studies have examined the effects of post-exercise ALC consumption on force [18,20,21,22,23,24,25,27,28]. McLeay et al. [23] evaluated maximal isometric, concentric and eccentric muscular contractions of the quadriceps femoris using an isokinetic dynamometer for both lower limbs using one lower limb as control. A significant difference between lower limbs was present post-treatment (exercised vs. non-exercised lower limb) up to 60 h post-exercise regarding maximal isometric tension, concentric and eccentric torque but no difference was observed between the ALC and the NO-ALC condition. Barnes et al. [18,27] in both studies evaluated isometric, concentric and eccentric contractions of the quadriceps muscles of both lower limbs using one lower limb as control. Isometric tension was measured at 75° of knee angle. Concentric and eccentric torque was measured at an angular speed of 30°/s. In both studies a decrease in performance was seen in both the ALC and NO-ALC groups over time in the exercised lower limb for all the evaluated measures (isometric and eccentric peak torques as well as for isometric, concentric and eccentric average peak torques). A greater decrease in performance was however seen in the ALC group in the first study [27] at 36h post-intervention (isometric and eccentric peak torques were reduced 39 and 44% compared to pre-exercise measures, respectively, with ALC whereas losses of 29 and 27% for the same measures in the NO-ALC group. Average peak torque was reduced by 41% (isometric), 43% (concentric) and 45% (eccentric) with ALC compared to 29, 32 and 26% with NO-ALC groups, respectively), while no differences were seen between 36 and 60h post-intervention. In the second study [18], except for average peak isometric torque, all measures were different between interventions with the greatest decrements observed in the ALC group. Greatest decreases in peak torque were observed at 36 h with losses of 12%, 28% and 19% occurring in the NO-ALC group for isometric, concentric and eccentric contractions, respectively. Peak torque loss was significantly larger in ALC with the same performance measures decreasing by 34%, 40% and 34%). Levitt et al. [22] measured peak torque for the knee extension exercise on each lower limb using an isokinetic dynamometer. The same assessment procedure used by Barnes et al. [18,27] was adopted. A reduction post-intervention was found for peak isometric, concentric and eccentric torque between the exercised and not-exercised lower limb, but no difference was found between the ALC and NO-ALC condition immediately post nor at 24 and 48 h post-intervention. Poulsen et al. [25] evaluated isokinetic muscle strength of the dominant knee extensors and non-dominant wrist flexors. No differences in isometric strength was observed immediately post, 4, 24 or 48 h post intervention, neither regarding time, when compared to baseline, neither regarding ALC condition. Murphy et al. [24] measured peak MVC of the knee extensors and found that a significant reduction compared to baseline was evident at all measured time points (2 and 16 h post-intervention), but no differences were present between the ALC and NO-ALC group. Haugvad et al. [21] have also measured isometric MVC of the knee extensors and the results reported by the authors showed stable values in all analysed conditions (Low ALC dose, High ALC dose and NO-ALC). A decrease immediately after performance and a recovery from immediately after to 12 and 24 h post intervention was seen in all groups, with no significant differences between trials. Clarkson et al. [20] measured isometric strength of the elbow flexors and the results are similar to those of Haugvad et al. with a reduction immediately post-exercise but no difference between conditions. The level of isometric strength returned to baseline 5 days post- intervention. Except for the studies of Barnes et al. no differences seem to be present following ALC consumption on force during recovery following RE. It has to be noted that the ALC dose provided by Barnes et al. is of 1g/kg, which is neither the minimum or maximum dose provided across the studies.

#### 3.2.2. Power

Four studies [19,24,28,30] included measures of power following ALC consumption and RE. Barnes et al. [19] have included measures of counter movement jump (CMJ) and horizontal power output (HPO). HPO did not vary neither over time neither regarding condition. CMJ instead showed a significant time x treatment effect, where a decrease in jump performance was observed after 24 and 48 h post-intervention only in the ALC group. However, the authors underline that the decrements seen in performance are trivial as the decrease in the jumping performance of the CMJ was a mean value of 12 cm. Murphy et al. [24] have included a measure of CMJ over time, where each participant was required to perform 10-maximal repeated CMJs. The results of Murphy et al. do not show any difference neither regarding time neither regarding condition. Levitt et al. [28] have included measures of vertical power, and similarly to Barnes et al., the reported measures of power show a time effect, with a reduction in vertical power output after 24 and 48 h post-intervention, but no effect regarding condition. Haugvad et al. [21] included a measure of squat jump performed without any counter movement on a force platform. Jump height was calculated for analysis. Jump height was reduced immediately after and 12 h post-intervention in all groups (low-ALC, high-ALC and NO-ALC condition). However, no difference between any group was present. ALC doesn’t seem to have an effect on power output, at least in the 48 h following its consumption.

#### 3.2.3. Muscular Endurance

Maximal isokinetic muscular endurance was calculated for the dominant knee extensors and non-dominant wrist in the study of Poulsen et al. [25] using an isokinetic dynamometer. Thirty maximal reciprocal movements were performed at a velocity of 180°/s without any rest interval. Subjects were instructed to exert maximal effort in every single movement and not to economise the muscle exertion. An endurance index was calculated defined as the mean-peak torque of the last five repetitions as a percentage of the mean-peak torque of the first five repetitions. The results obtained by the authors show no differences in muscular endurance 4, 24 and 48h after treatment neither after ALC intoxication neither in the NO-ALC group. No changes were evident neither in the leg extensors neither in the wrist flexors or between the endurance index for both conditions for both muscle groups. The results were also stratified according to gender and similar findings were achieved.

#### 3.2.4. Soreness

Five studies included measures of soreness [18,20,22,23,28]. Barnes et al. [18] evaluated soreness by asking each participant at different time points their levels of soreness by giving a value from 0 (no pain) to 10 (worst possible pain). Soreness was rated while stepping up (concentric muscular contraction) onto a 40 cm box and lowering into a squatting position. Clarkson et al. [20] evaluated soreness by questionnaire, measured for the forearm flexor muscles, using a scale of 1 to 10. Levitt et al. [22] measured soreness applying on the vastuslateralis, in three different points along the muscle belly, a pressure of 35N. Each participant rated the pain giving a value from 0 to 10. In a subsequent study Levitt et al. [28] evaluated pain by asking the participants to self-report their level of pain using a scale from 0 to 5. McLeay et al. [23] used the same protocol as above described in the study of Barnes et al. [18]. All the retrieved records show a time effect for muscle soreness related to the intervention protocol, with increases over a period of 24 and 48 h after the training intervention, with no differences between the ALC and the NO-ALC condition. The results indicate that RT is a factor responsible to increase muscle soreness between 24 and 48 h post training, whereas ALC consumption is not.

#### 3.2.5. Rate of Perceived Exertion

Rates of perceived exertion were measured in three of the retrieved studies [19,24,26]. The study of Vingren [26], was the only one evaluating RPE before and after a single session of static RE. Barnes and Murphy [19,24] evaluated RPE before and after a rugby match. In particular Murphy et al. evaluated RPE after a competitive rugby league game, whereas Barnes et al. after a simulated rugby match. The results of Vingren and Murphy highlight a significant time effect, with increases of RPE after the RT and the rugby league game, but no significant differences between the RPE of the ALC or the NO-ALC groups. The results of Barnes et al. are in line to those of the previous authors regarding the time effect of RPE following the exercising protocol, however a difference was shown between conditions. The ALC group reported lower levels of RPE at the end of the third quarter of the simulated game, compared to the NO-ALC group at the same time measurement. Despite the significant results, the mean difference between the two conditions is very small (ALC=15.2 ± 1.6; NO-ALC=16.5 ± 1.2) and not present at any other time point.

### 3.3. Cognitive Function

Cognitive function was assessed only in the study of Murphy et al. [24] through a modified version of the Stroop test. This test of cognitive function was a computer-based program requiring subjects to react to repeated color and word stimuli. The program analyzed response time and accuracy for congruent and incongruent stimuli. Measures of cognitive function were recorded before, immediately post, 2 and 16 h intervention. The results provided by the authors show no difference over time for cognition test time, congruent reaction time, or incongruent reaction time. However, the time required to complete the cognition test significantly increased in the ALC compared to the NO-ALC group and a large ES was shown for increased cognition test time, congruent and incongruent reaction time in the ALC group compared to the NO-ALC group. The findings indicate that ALC consumption impairs cognitive function during recovery, which may be a negative factor in sports where decision making processes, speed and quality of responses to visual stimuli (especially team sports) are essential.

## 4. Discussion

By evaluating the effects of alcohol consumption on recovery following RE from biological, physical and cognitive perspective, we have been able to provide a comprehensive description of the multifactorial nature of alcohol consumption. Indeed, alcohol consumption is a common occurrence in the general population on global scale and it is a phenomenon that has not been explored in depth when it comes to post-exercise recovery, even more so in RE post-exercise recovery. The main findings highlight that ALC cannot be considered as a modulating factor for the majority of the retrieved biological measures. In fact, creatine kinase, heart rate, lactate, blood glucose, estradiol, sexual hormone binding globulin, leukocytes and cytokines, C-reactive protein and calcium do not seem to be modified following ALC consumption during the acute recovery phase post-resistance exercise. Only cortisol levels seem to be increased, conversely testosterone, plasma amino acids, and rates of muscle protein synthesis decreased. When considering the retrieved physical measures force, power, muscular endurance, soreness and rate of perceived exertion also seem to be unmodified following alcohol consumption during recovery. The general findings therefore highlight that muscle function is not altered by alcohol consumption following exercise bouts, however the altered endocrinological asset regarding cortisol and testosterone and the consequent suppressed rates of muscular protein synthesis and reduced circulating levels of amino acids, suggest that long-term muscular adaptations could be impaired.

A trend of heart rate increase following the exercise intervention has been detected, however no difference was shown between the ALC and the NO-ALC condition. This conclusion raises different concerns since alcohol acts as a diuretic and it contributes to faster elimination of water content from the bloodstream, leading to increased viscous blood plasma which is harder to pump and deliver to the body tissues [31]. The heart has to adapt to these conditions to increase the cardiac output. There seems to be a dose response relationship between the alcohol consumption and heart rate i.e., there is a positive correlation between alcohol consumption and heart rate response [32]. Consequently, this has the potential to alter individual RPE [33]. This latter parameter also seems to not be influenced by alcohol consumption. Only one of the retrieved studies has shown there was a difference between the ALC and NO-ALC group with the ALC group showing less perceived exertion compared to the NO-ALC group.

As previously stated alcohol acts as a diuretic and thus can explain why in the study of Murphy at al [24] the ALC group had an increased total volume output overnight when compared to the NO-ALC group.

One of the most interesting findings of this review was found in the study by Parr et al. [9] who demonstrated that blood glucose is affected by CHO but not ALC during recovery after RE. This is in alignment with findings of Lustig [34] who claims that toxic effects of alcohol are very similar to excessive sugar exposure mainly for its fructose content. Even though fructose does not show the same acute toxic effects of ethanol, it encompasses all the chronic hazardous effects on long-term health [34].

Creatine kinase was also unmodified by ALC consumption. Such enzyme which is present in the muscles, when detectable in the peripheral circulation, is commonly used as a measure of muscle damage [8]. None of the authors which reported measures of CK showed differences between groups, instead correlations were established between the ALC and the NO-ALC condition. ALC cannot be considered as a modulating factor for CK following RE and the increases of CK shown are the result of muscle damage following the exercise bouts. Neither leukocytes nor cytokines seem to be changed following alcohol consumption, which means that the inflammatory response is not modulated by alcohol consumption. Such is a controversial finding because as reported by different authors [35,36] alcohol abuse not only increases inflammation but also alters the immune function of the body. Probably healthy individuals, who regularly exercise, as those included in each study of this review, do not express altered inflammatory or immune function following a single acute alcohol intoxication. Same trend is shown by CRP, which confirms that muscle damage and inflammation are not dependent, in the analyzed population, from the ingestion of alcohol [24]. Such findings may also explain why perceived soreness was not different between the ALC and NO-ALC groups analyzed.

Cortisol and testosterone levels during post RE when compared between ALC and NO-ALC groups appear to be altered. On average, the participants who consumed ALC expressed higher levels of cortisol and lower levels of testosterone in comparison to the NO-ALC group. Decreased levels of testosterone and increased levels of cortisol are suggested to be indicative for a disturbance in the anabolic-catabolic balance, which likely leads to decreased recovery and therefore, decreased levels of performance [24,37]. When present in excessive levels, cortisol is an overall catabolic hormone, which decreases lean body and muscle mass and increases energy expenditure [38]. Conversely, testosterone is an anabolic hormone, which may also explain why in the study of Parr et al. mTOR phosphorylation in the NO-ALC group was higher than the ALC-CHO (76%) and ALC-PRO (54%) group at 2 and 8 h post-exercise. In addition, also rates of muscle protein synthesis were higher in the NO-ALC group when compared to those who ingested ALC. However, muscle protein synthesis may also appear decreased because of the decreased plasma levels of AA showed following ALC consumption. These findings can have major implications with regards to the recovery and performance of both non athletes and professional athletes. An acute bout of vigorous RE can result in a transient increase in protein turnover and until feeding, protein balance remains negative [39,40]. Protein ingestion post exercise enhances muscle protein synthesis and net protein balance [41] by increasing myofibrillar protein fraction with RE [42], but as seen in the study of Parr et al. alcohol ingestion after RE has the ability to disrupt this process. Beyond physical aspects, decreased protein synthesis leads to impaired long-term memory in humans [43,44,45], which can be particularly important in professional athletes who have many cognitive demands with respect to both short and long term memory [17].

In regards to measures of force only study of Barnes et al. [18] showed that following ALC consumption the levels of isometric, concentric and eccentric torque decreased, while other studies in this review that measured force production showed no differences between the ALC and NO-ALC groups during recovery following RE. As depicted in the results section, with respect to muscle function and force, only study by Barnes et al. has shown that moderate consumption of alcohol can amplify the loss of force associated with strenuous eccentric exercise [18]. This particular study detected significant decrements in average peak isometric, concentric and eccentric torques at 36 h post-exercise [18]. Clearly more research is needed since the outcomes among the mentioned studies are quite distinct. All measures of force were assessed from 2 h to 48 h post RT or ALC ingestion and the measures all appear decreased because of the exercise performed. The retrieved measures of performance returned to baseline within 2 days following both ALC consumption and the exercise bouts. Same trend is shown for the other two measures of performance retrieved: power and muscular endurance which decreased following the exercise bouts in both groups with no difference between those who consumed ALC and those who did not.

Several limitations have been encountered during the realization of this manuscript. Firstly, a very limited body of evidence was present within each screened database, on the topic of alcohol consumption following bouts of RE, therefore it is not possible to consider such review comprehensive and definitive. Few studies have evaluated in depth biological measures of protein synthesis or specific markers of muscular function. Other important limitation is the timely evaluation of each study. Each included measure was evaluated in a timeframe ranging from 2 h to 48h post exercise or alcohol consumption. Therefore, only acute modifications were evaluated and it was not possible to consider hormonal fluctuations beyond 2 days and their relative effects. Lastly, the total sample size of each study was small ranging between 8 and 19 participants.

## 5. Conclusions

Alcohol consumption following resistance exercise doesn’t seem to affect the majority of the retrieved biological and physical measures. However, levels of cortisol were increased, and levels testosterone and rates of muscle protein synthesis were decreased, which indicates that long term muscular adaptations could be impaired if alcohol consumption during recovery is consistent. Muscle function doesn’t seem to be influenced by alcohol consumption during recovery. Studies with larger cohorts evaluating the effects of alcohol consumption during recovery following resistance exercise are needed to further understand the long-term effects of alcohol ingestion.

## Figures and Tables

**Table 1:** Descriptive characteristics of the retrieved studies.

Author [Ref] (Year)	*n*	Aim	Alcohol (dose)	Mix	Administration Time	Measures	Resistance Training	Comparator	Outcomes (Compared to Comparator)
Barnes et al. [27] (2010)	12	Evaluate if ALC interacts with damaged muscles.	1g/kg	37.5% ALC/volume; Smirnoff Vodka in orange juice (ratio 3.2:1)	A beverage was consumed every 15 min over a total time of 90 min.	-Strength. -Peak and averaged torque.	300 maximal eccentric contractions of the quadriceps muscles of one lower limb at an angular speed of 30°/s.	Cross-Over	No differences in acute performance measures. Decreased performance was seen after 36h following ingestion.
Barnes et al. [18] (2010)	11	To compare the effects of post-exercise ALC ingestion with that of an isocaloricnon-ALC beverage on changes in muscle performance.	1g/kg	37.5% ALC/volume; Smirnoff Vodka in orange juice (ratio 3.2:1).	A beverage was consumed every 15 min over a total time of 90 min.	-Soreness -Peak and averaged torque -CK	300 maximal eccentric contractions of the quadriceps muscles of one lower limb at an angular speed of 30°/s.	Cross-Over	Peak concentric, eccentric were lower in the ALC group. No differences in CK and soreness
Barnes et al. [19] (2012)	10	To investigate the effects of post-game ALC consumption on whole-body, sport-specific performance.	1g/kg	37.5% ALC/volume; Smirnoff Vodka in orange juice (ratio 3.2:1).	A beverage was consumed every 15 min over a total time of 90 min.	-HR -Lactate -RPE -CMJ -HPO -CK -Cortisol and Testosterone -Leukocytes	BURST Protocol (intense 20-m shuttle run with 180° turns)	Cross-Over	HR and Lactate showed no difference. RPE varied significantly. Differences in CMJ but not in HPO were present. No differences in the leukocyte count. CK was higher in the ALC group only after 48h. Testosterone did not show any differences. Cortisol was higher in the ALC group after 36h.
Clarkson et al. [20] (1990)	10	Assess the effect of acute ALC ingestion on muscle indicators.	0.8g/kg	Vodka 40% with orange juice (ratio 1:1)	Single dose.	-CK -Soreness -Isometric strength	50 repetitions at a lat-pulley.	Cross-Over.	No difference in CK. No difference in soreness. No difference in strength.
Haugvad et al. [21] (2014)	9	Investigate the effects of ethanol on recovery of muscle function after RT.	-Low dose 0.6 or 0.7 g/kg -High dose 1.2 or 1.4 g/kg	40% ethanol/volume, Absolut vodka diluted with 200-mL sugar-free lemonade(raspberry flavour) and water to a total of 1.5 L	The beverage was consumed in about 90 min.	-MVC -Power -Cortisol and Testosterone -SHBG -CK -Leukocytes.	Squats, lower limb presses, and bilateral knee extensions were performed in 4 sets with a load of 8RM with 2 min rest.	Cross-Over	MVC was decreased after the ALC trial 12h training. MVC normalized in both groups after 24h. No difference in Jump performance; Cortisol was higher at 12 at 24h in the high dose group. Neither testosterone or SHBG were influenced by ALC. Free testosterone was lower in the high dose group at 12 and 24h. No differences in the CK for any group. No differences in leukocytes.
Levitt et al. [22] (2017)	13	The effect of acute ALC consumption on muscular recovery process.	1.09 g/kg	The ALC was diluted to 15% *v/v* in an artificially sweetened beverage.	The beverage volume was split into 10 equal portions; one portion was administered each minute over a 10min ingestion period.	-TNF-α -Il-1β -Il-6 -Il-8 -Il-10 -Soreness -Isometric, concentric and eccentric torque -CK	300 maximal single-lower limb eccentric leg extensions.	Cross-Over	No difference in soreness. No difference in strength. No difference in CK. No difference in any cytokine.
Levitt et al. [28] (2018)	10	To investigate the effect of ALC consumed after heavy eccentric resistance exercise on measures of muscle power.	1.09 g/kg	Smirnoff 40% ALC Vodka diluted to 15% *v/v* in an artificially sweetened beverage.	The beverage was split into 10 equal portions and one portion consumed every 3 min during the 30-min beverage ingestion period.	-Soreness -Peak power -Peak force -Jump height	4 sets of 10 repetitions at 110% of concentric 1RM; 3 min passive rest in between sets	Cross-Over	No differences were found in peak power nor peak force or jump height. No differences were found in soreness measures.
McLeay et al. [23] (2017)	8	To investigate the effects of ALC consumption on recovery of muscle force.	0.88 g/kg	37.5% ALC/volume; Smirnoff Vodka in orange juice	Six drinks were consumed every 15 min over 1.5 hr.	-CK -Soreness -Isometric, concentric and eccentric torque.	300 maximal single-lower limb eccentric leg extensions through a 60° ROM at an angular speed of 30°/s.	Cross-Over	No difference in isometric, concentric and eccentric torque. No difference in CK. No difference in muscle soreness.
Murphy et al. [24] (2013)	9	To evaluate the effects of ALC ingestion on lower-body strength and power and physiological and cognitive recovery	1g/kg	37.5% ALC/volume; Smirnoff Vodka in orange juice (ratio 3.2:1).	An equal volume of beverage was consumed every 20 min over a total time of 150 min	-RPE -CMJ -MVC -Urine -CK -CRP - Cortisol and Testosterone -Cognitive function	Rugby match	Cross-Over	No difference in RPE. No differences in CMJ and MVC. No difference in CK and CRP. No difference in testosterone. Large effect size for cortisol increase after 16h in the ALC group. Larger urine volume after night in the ALC group. Decreased cognitive function was observed in the ALC group.
Parr et al. [9] (2014)	8	Evaluate the effect of ALC intake on rates of myofibrillar protein synthesis following strenuous exercise	-1.5g/kg with CHO -1.5g/kg with PRO	Vodka and Orange juice (ratio 1:4)	6 equal volumes were consumed during a 3 h period.	-Biopsy -Blood glucose -Plasma AA concentration -Intracellular signalling proteins	-8 × 5 at ,80% of 1RM -10 × 30 s high intensity intervals at 110% of PPO; 3 min rest between sets	Cross-Over	Blood ALC was higher in the CHO compared to the PRO group after 6 and 8h after consumption. Blood Glucose was higher in the ALC-CHO group after 5h.AA (EEA and BCAA) were lower in the ALC groups compared to the no ALC group.mTOR phosphorylation was higher in the no ALC group at 2 and 8h post exercise. p70S6Kphosphorylation was higher in the no ALC and the ALC-PRO group at 8h post exercise. Muscle protein synthesis was greater in the No ALC group than the ALC-PRO, which was greater than the ALC-CHO group.
Poulsen et al. [25] (2007)	19	Evaluate acute ALC intoxication on skeletal muscle function	1.5 g/L	ALC 96% with orange juice (ratio 1:4)	5 doses with intervals of 1h each.	-CK -Ca^2+^ -Strength -Endurance	MVC Isokinetic endurance and isometric knee extensors (30 extensions at a velocity of 180°/s)	Cross-Over	No differences in strength and endurance. No differences in CK. Small reduction in Ca^2+^ only in the ALC group.
Vingren et al. [26] (2013)	8	To examine the testosterone bioavailability and the anabolic endocrine milieu in response to acute ethanol ingestion	1.09 g/kg	ALC was diluted to a concentration of 19% *v/v* absolute ethanol in an artificially sweetened and calorie-free beverage	The participants drank 1/10 of the drink each minute during a 10-min ingestion period.	-HR -RPE -Testosterone -SHBG -Lactate -Cortisol -Estradiol	6 × 10squats starting at 80% of 1 RM and 2 min of rest between sets.	Cross-Over	No difference in HR, RPE and lactate. Serum testosterone and free testosterone was higher for ALC at 300min post exercise. FAI was higher in the ALC group. No difference in cortisol levels. No differences in estradiol.
	Tot. 127		Mean 1.1g/kg						

N= Number of participants; g/L= grams per liter; g/kg= grams per kilogram; ALC= Alcohol; CK= Creatine kinase; Ca^2+^= Calcium; MVC= Maximum voluntary contraction; ROM= Range of movement; HR= Heart rate; RPE= Rating of perceived exertion; CMJ= Counter movement jump; HPO= Horizontal power output; RE= Resistance exercise; SHBG: Sex hormone-binding globulin; RM= Repetition maximum; CRP= C-reactive protein; CHO= carbohydrate; PRO= Protein.; AA= Amino Acids; PPO= Peak power output; EAA = Essential amino acids; BCAA= Branched Chain amino acids; FAI= Free androgen index.

## Supplementary Materials

Supplementary materials can be found at https://www.mdpi.com/2411-5142/4/3/41/s1.
