# Supplementary material for: The Effects of Alcohol Consumption on Recovery Following Resistance Exercise: A Systematic Review
Source: J Funct Morphol Kinesiol. 2019 Jun 26;4(3):41. doi: 10.3390/jfmk4030041 (PMC7739274; doi:10.3390/jfmk4030041)
Supplement: Supplementary file 1 [file jfmk-04-00041-s001.pdf]

Researcher One

Author	year	Reporting (_/11)	External Validity (_/3)	Internal Validity – Confounding (_/7)	Internal Validity – Bias (_/6)	Power (_/5)	Total score (_/32)
**Original study**							
Barnes et al [1]	2010	7	0	4	0	4	15
Barnes et al [2]	2010	7	0	4	1	4	16
Barnes et al [3]	2012	8	0	5	0	4	17
Clarkson et al [4]	1990	5	0	4	0	3	12
Haugvad et al [5]	2014	7	1	4	2	5	19
Levitt et al [6]	2017	6	0	4	4	5	19
Levitt et al [7]	2018	8	0	3	1	4	16
McLeay et al [8]	2017	9	0	3	2	4	18
Murphy et al [9]	2013	7	0	4	3	4	18
Parr et al [10]	2014	7	0	4	2	5	18
Poulsen et al [11]	2007	7	0	6	0	5	18
Vingreen et al [12]	2013	6	0	5	2	5	18

Researcher Two

Author	year	Reporting (_/11)	External Validity (_/3)	Internal Validity – Confounding (_/7)	Internal Validity – Bias (_/6)	Power (_/5)	Total score (_/32)
**Original study**							
Barnes et al [1]	2010	9	0	4	3	3	19
Barnes et al [2]	2010	8	1	3	4	4	20
Barnes et al [3]	2012	8	0	4	4	3	19
Clarkson et al [4]	1990	6	0	4	2	2	14
Haugvad et al [5]	2014	11	2	3	4	5	25
Levitt et al [6]	2017	8	2	4	6	4	24
Levitt et al [7]	2018	9	3	4	4	4	24
McLeay et al [8]	2017	8	0	5	5	4	22
Murphy et al [9]	2013	7	0	4	3	4	18
Parr et al [10]	2014	9	0	5	5	4	23
Poulsen et al [11]	2007	9	0	6	5	4	24
Vingreen et al [12]	2013	9	1	7	6	5	28

Study	High quality (23-32)	Moderate quality (19-22)	Lower quality (16-18)	Poor quality (<14)
**Barnes et al [1][26]**			17	
**Barnes et al [2][17]**			18	
**Barnes et al [3][18]**			18	
**Clarkson et al [4][19]**				13
**Haugvad et al [5][20]**		22		
**Levitt et al [6][21]**		22		
**Levitt et al [7][27]**		20		
**McLeay et al [8][22]**		20		
**Murphy et al [9][23]**	23			
**Parr et al [10][9]**		21		
**Poulsen et al [11][24]**		21		
**Vingreen et al [12][25]**	25			

References

1. Barnes, M.J., T. Mundel, and S.R. Stannard, Post-exercise alcohol ingestion exacerbates eccentric-exercise induced losses in performance. *Eur J Appl Physiol***2010**, *108*, 1009–1014.
2. Barnes, M.J.; Mundel, T.; Stannard,S.R. Acute alcohol consumption aggravates the decline in muscle performance following strenuous eccentric exercise. *J. Sci Med. Sport***2010**, *13*, 189–193.
3. Barnes, M.J.; Mundel, T.;Stannard, S.R. The effects of acute alcohol consumption on recovery from a simulated rugby match. *J. Sports Sci***2012**, *30*, 295–304.
4. Clarkson, P.M.; Reichsman, F. The effect of ethanol on exercise-induced muscle damage. *J. Stud. Alcohol* **1990**, *51*, 19-23.
5. Haugvad, A., et al.Ethanol Does Not Delay Muscle Recovery, but Decreases the Testosterone: Cortisol Ratio. *Med. Sci Sports Exerc.***2014**, *46*, 2175–2183.
6. Levitt, D.E.; Hiu-Ying, L.; Duplanty, A.A.; McFarlin, B.K.; Hill, D.W.; Vingreen, J.L. Effect of alcohol after muscle-damaging resistance exercise on muscular performance recovery and inflammatory capacity in women. *Eur. J. Appl. Physiol*.**2017**, *117*, 1195–1206.
7. Levitt, D.E.; Idemudia, N.O.; Cregar, C.M.; Duplanty, A.A.; Hill, D.W.; Vingreen, J.L. Alcohol after Resistance Exercise Does not Affect Muscle Power Recovery.*J. Strength Cond. Res.* 2018,DOI: 10.1519/JSC.0000000000002455 .
8. McLeay, Y.; Stannard, S.R.; Mundel, L.; Foskett, A.; Barnes, M. Effect of Alcohol Consumption on Recovery From Eccentric Exercise Induced Muscle Damage in Females. *Int. J. Sport. Nutr. Exerc. Metab.***2017**, *27*, 115–121.
9. Murphy, A.P.; Snape, A.E.; Minett. G.M.; Skein, M.; Duffield, R. The effect of post-match alcohol ingestion on recovery from competitive rugby league matches. *J. Strength Cond. Res.***2013**, *27*, 1304–1312.
10. Parr, E.B.; Camera, D.M.; Areta, J. L.; Burke, L.M.; Phillps, S.M.; Hawley, J.A.; Coffey, V.G. Alcohol ingestion impairs maximal post-exercise rates of myofibrillar protein synthesis following a single bout of concurrent training. *PLoS ONE* 2014,**9**, e88384.
11. Poulsen, M.B.; Jakobsen, J. Aagaard, N.K.; Andersen, H.; Motor performance during and following acute alcohol intoxication in healthy non-alcoholic subjects. *Eur. J. Appl. Physiol.***2007**, *101*, 513–523.
12. Vingren, J.L.; Hill, D.W.; Buddhadev, H.; Duplanty, A. Postresistance exercise ethanol ingestion and acute testosterone bioavailability. *Med. Sci Sports Exerc* **2013**, *45*, 1825–1832.
